# 

**Published:** 1887-09

**Authors:** 


### TABLE OF CONTENTS.

### Original and Selected Articles.

Dynamite, by E. van Goidtsnoven, Atlanta, Georgia ... 353
Man an Object of Zoology, by S. W. Stiles, M. D, Georgia ... 357
Aseptic Dressing of Penetrating and Punctured Wounds, by O. Grothan, M. D., Scotia, Nebraska ... 359
Fracture of Cranium; Hernia Cerebri; Recovery, by William Henry, M. D., of Harmon, Illinois ... 362
The Tribulatiors of a Medical Student, by "Quigley," M. D ... 363
Surgical Aphorisms, by J. J. Berry, M.D., Portsmouth, New Hampshire ... 366
Prognosis of Paralysis Occurring in Children Determined by Electric Examination, by E. C. Smith, B. Sc., M. D., Richmond, Va ... 368
An Anodyne in Use for Vesical Irritation ... 369

### Abstracts and Gleanings.

Brain Surgery ... 370
A Speedy and Sometimes Successful Method of Treating Hay Fever ... 372
Lactation and Electricity ... 373
A Novel and Painless Corn Salve ... 374
A New Operation for Hernia; Paracoto in Disentery ... 375
An Unusually Rapid Case of Graves' Disease ... 376
Boracic Acid in the Treatment of Leuchorrhœa; Treatment of Pulmonary Tuberculosis by Subcutaneous Injections of Eucalyptol ... 377
Tirections for Vaccinating ... 378
Dreatment of Grave Epistaxis; Veterinary Medicine—Equine Founder ... 379
Dysentery; A Young Physician ... 380
Lantanine, a Qunine Substitute; Resorcin Inoculations in Phlegmon; Passage of a Large Foreign Body Through the Alimentary Canal of an Infant; A Specific for Diabetes 381
Nettle Juice; Possible Substitutes for Cocaine in Medicine; Prolapse of Rectum; Atropine in Carbolic Acid; Spasmodic Croup in Children ... 382

### Scientific Items.

Military Dogs ... 383
A New Light for Instantaneous Photographs; Do not Eat Raw Eggs; A Lense ... 384

### Practical Notes and Formulæ.

For Cholera, Diarrhœa, etc.; For Catarrhal Conjunctivit s; Syrup Doveri; For Clergyman's Sore Throat; Nerve Tonic in Opium Cases; Singultus ... 385
Puerperal Convulsions; Diphtheria; Diarrhœa 386
Scabies; Constipation; A Good Cholagogue Pill; For Summer Diarrhœa; For Vomiting of Cholera Infantum; Neuralgia ... 387

### Editorials and Miscellaneous.

To Our Subscribers; Editorial Brevities ... 388
The Dissection Bill ... 389
International Medical Congress; Typho-Malarial Fever ... 390
Dr. Bryce and the Virginia Medical College ... 391
Perforation of the Appendix Vermiformis; Books and Pamphlets Received ... 392
Receipted; Special Notes ... 395

